# Comparison of postoperative outcomes following multidetector computed tomography based vs transesophageal echocardiography based annulus sizing for transcatheter aortic valve replacement: A systematic review and meta‐analysis

**DOI:** 10.1111/echo.14684

**Published:** 2020-09-23

**Authors:** Guozhang Tang, Qifeng Lv, Xiangqin He

**Affiliations:** ^1^ Department of Echocardiography The Affiliated Hospital of Qingdao University Qingdao China

**Keywords:** aortic valve, complications, computed tomography, echocardiography, transcatheter aortic valve replacement

## Abstract

**Background:**

The purpose of this paper was to evaluate the difference in postoperative outcomes following multidetector computed tomography (MDCT) and transesophageal echocardiography (TEE)‐based annulus sizing for transcatheter aortic valve replacement (TAVR).

**Methods:**

Electronic search of PubMed, Biomed Central, Scopus, and Google Scholar databases was conducted until August 15, 2019. We included all types of studies comparing MDCT‐based annulus sizing with TEE‐based annulus sizing and assessing paravalvular regurgitation (PVR). Data were summarized using the Mantel‐Haenszel odds ratio (OR) with 95% confidence intervals (CI).

**Results:**

A total of six studies were included. Pooled analysis of 431 participants in the MDCT group and 509 participants in the TEE group demonstrated that MDCT‐based annulus sizing is associated with a significantly lower incidence of more than moderate PVR as compared to 2DTEE‐based sizing (OR: 0.31, 95% CI: 0.18‐0.54, *P* < .0001; *I* ^2^ = 0%). There was no statistical difference in annulus rupture (OR: 0.57, 95% CI: 0.12‐2.66, *P* = .91; *I* ^2^ = 0%), procedural mortality (OR: 0.97, 95% CI: 0.19‐4.86, *P* = .97; I^2^ = 0%), and 30‐day mortality (OR: 0.63, 95% CI: 0.26‐1.50, *P* = .29; *I* ^2^ = 0%) with MDCT or 2DTEE‐based annulus sizing. Compared with 3DTEE, the incidence of PVR in the MDCT group was lower, but there was no statistical difference in 30‐day mortality.

**Conclusion:**

Use of MDCT in comparison with 2DTEE is associated with significantly lower incidence of more than moderate PVR after TAVR. There seems to be no difference in annulus rupture and 30‐day mortality with either imaging modality.

## 1 INTRODUCTION

Transcatheter aortic valve replacement (TAVR) is an effective therapeutic modality in managing patients with severe aortic stenosis.^1^ Though a highly successful procedure, complications like paravalvular aortic regurgitation (PVR) can be seen in up to 38% of patients undergoing TAVR.^1^, ^2^ The occurrence of PVR consequently results in poor clinical outcomes and a significant increase in mortality. Tamburino et al^3^ reported PVR to be an independent predictor of mortality between 30 days and 1 year, in a sample of 663 patients. The authors observed a fourfold increased risk of mortality in patients demonstrating more than moderate postprocedural PVR.^3^

Incongruous sizing of the aortic annulus resulting in inappropriate valve selection is a major reason for postoperative PVR. The junctional nadirs of the aortic leaflets at the distal part of the left ventricular outflow tract form a virtual ring that is regarded as the aortic annulus during TAVR.^4^ In the absence of a discrete anatomical structure, accurate assessment of the annulus via appropriate imaging is critical in preventing PVR. On the other hand, oversizing of the prosthetic valve can lead to significant complications like annulus rupture, coronary obstruction, and conduction disturbances.^5^

Traditionally, two‐dimensional (2D) transesophageal echocardiography (TEE) has been used for evaluating annulus size for TAVR.^6^ However, it is increasingly recognized that 2DTEE may not accurately measure the oval three‐dimensional (3D) annulus structure and considerable sizing variations may occur depending upon the axis of orientation.^4^, ^7^ The use of 3DTEE has been described to overcome the limitations of 2DTEE with significantly higher annulus diameters achieved with exclusive use of 3DTEE for valvular sizing.^8^ Over the last decade, multidetector computed tomography (MDCT) has been increasingly used for annulus sizing before TAVR, as it provides a detailed understanding of the valvular anatomy with a superior spatial resolution.^9^ Studies have demonstrated that annulus measurements with 2DTEE frequently result in valve undersizing as compared to MDCT‐based measurements.^10^ On the other hand, a recent meta‐analysis by Rong et al^11^ has shown that measurements by 3DTEE may be comparable to that of MDCT and may lead to reduced contrast exposure. While multiple studies have compared differences in annulus sizing with TEE and MDCT,^10^, ^12^, ^13^ evidence on the effect of imaging modality on the postoperative outcomes has not been summarized to date. Therefore, the purpose of this systematic review and meta‐analysis was to evaluate the difference in postoperative outcomes following MDCT and TEE‐based annulus sizing for TAVR.

## 2 METHODS

The guidelines of the PRISMA statement (Preferred Reporting Items for Systematic Reviews and Meta‐analyses)^14^ and the Cochrane Handbook for Systematic Reviews of Intervention were followed during the conduct of this review.^15^ The research question to be answered was the following: Does using MDCT‐based annulus sizing in TAVR associate with a lower incidence of PVR and improved clinical outcomes as compared to TEE‐based measurements?

### 2.1 Search strategy

A computerized literature search of PubMed, Biomed Central, Scopus, and Google Scholar databases was carried out. The last literature search was conducted on August 15, 2019. Two independent reviewers performed the electronic search using the following keywords: “Multidetector Computed Tomography,” “Computed Tomography,” “MDCT,” “Transesophageal Echocardiography,” “Echocardiography,” “TEE,” “transcatheter aortic valve replacement,” “transcatheter aortic valve implantation,” “paravalvular regurgitation,” “paravalvular leak,” and "clinical outcomes." The search strategy and results of the PubMed search are presented in Table S1. We also performed a manual search of references of included studies and review articles on the subject for identification of any additional studies. After assessing the studies by their titles and abstracts, full texts of selected articles were retrieved. Both the reviewers assessed individual studies based on inclusion criteria. Disagreements, if any, were resolved by mutual agreement.

### 2.2 Inclusion criteria and outcomes

Utilizing the PICOS (Population, Intervention, Comparison, Outcome, and Study design) outline, we included all types of studies conducted on patients undergoing TAVR (*Population*), comparing MDCT‐based annulus sizing (*Intervention*) with TEE‐based annulus sizing (*Comparison*) and assessing PVR and other clinical outcomes (*Outcomes*). At the protocol stage, we aimed to include studies comparing both 2DTEE and 3DTEE with MDCT for annulus valve sizing in TAVR patients. Studies comparing MDCT and TEE‐based annulus measurements on the same group of patients were excluded. We also excluded single‐arm studies, case reports, review articles, and non‐English language studies.

Using an abstraction form, two reviewers retrieved data from selected studies. The following details were sourced: Authors, publication year, sample size, inclusion/exclusion criteria, baseline characteristics, MDCT and TEE protocol, PVR, and any other clinical outcomes. The primary outcome was the incidence of moderate‐severe PVR. Secondary outcomes were the incidence of annulus rupture, procedural mortality, and 30‐day mortality.

### 2.3 Risk of bias assessment

Retrospective cohort studies were analyzed using the risk of bias assessment tool for nonrandomized studies (RoBANS).^16^ Studies were rated as low risk, high risk, or unclear risk of bias for the following: selection of participants, confounding variables, intervention measurements, blinding of outcome assessment, incomplete outcome data, selective outcome reporting. Quality of randomized control trials (RCTs) was assessed using the "Cochrane Collaboration risk assessment tool".^17^ Studies were rated as low risk, high risk, or unclear risk of bias for the following: random sequence generation, allocation concealment, blinding of participants and personnel, blinding of outcome assessment, incomplete outcome data, selective reporting, and other biases.

### 2.4 Statistical analysis

Because of significant heterogeneity among studies, a random‐effects model was used to calculate the pooled effect size. Categorical data were summarized using the Mantel‐Haenszel odds ratio (OR) with 95% confidence intervals (CI). Heterogeneity was calculated using the *I* ^2^ statistic. *I* ^2^ values of 25%‐50% represented low, values of 50%‐75% represented medium, and more than 75% represented substantial heterogeneity. A sensitivity analysis was carried out to assess the influence of each study on the pooled effect size. The software “Review Manager” (RevMan, version 5.3; Nordic Cochrane Centre [Cochrane Collaboration], Copenhagen, Denmark; 2014) was used for the meta‐analysis. Publication bias was not assessed using funnel plots as there were less than 10 studies in our analysis.^15^

## 3 RESULTS

**Figure 1:**
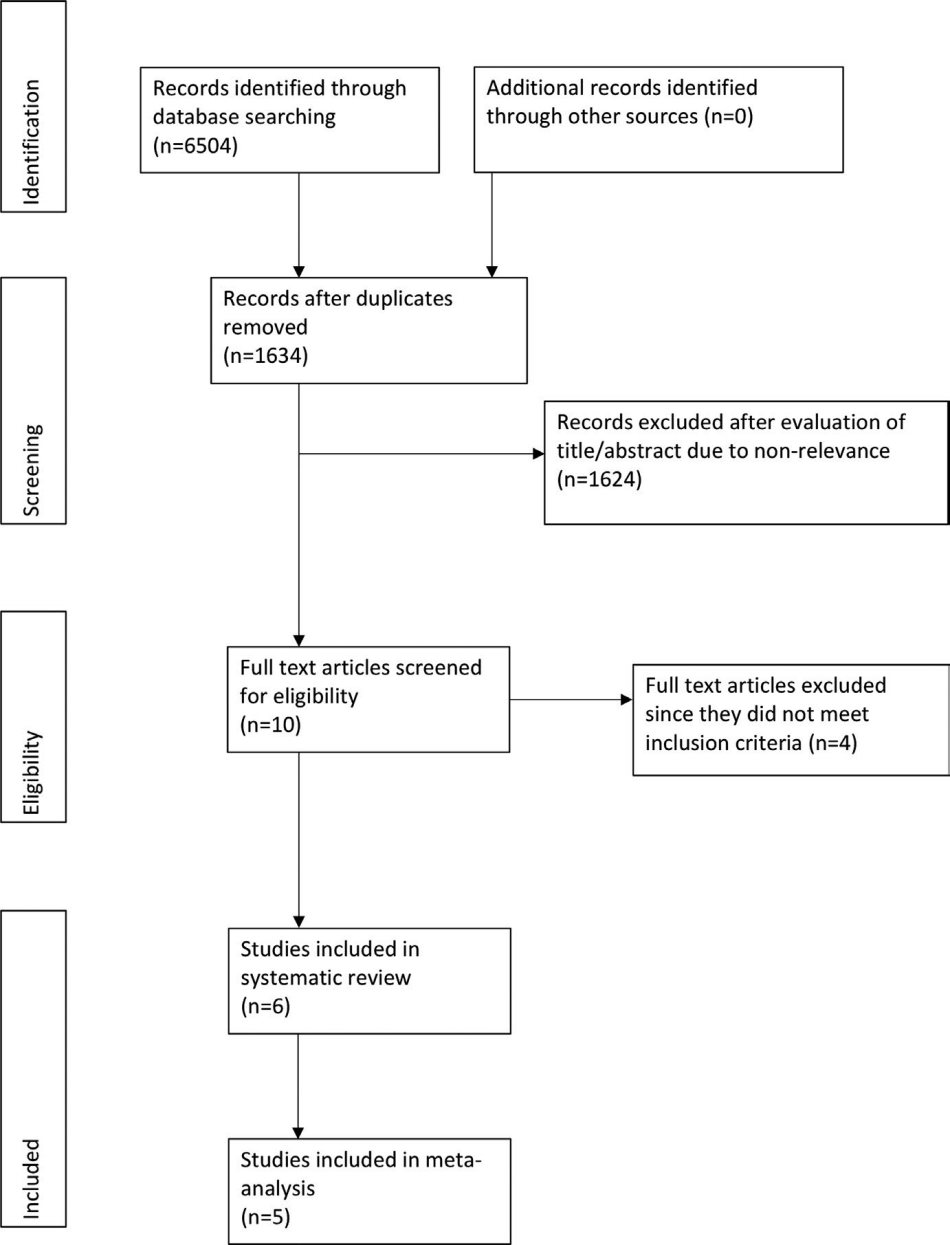
Systematic review and meta‐analysis flow diagram

The study flowchart is presented in Figure 1. Four studies were excluded after full‐text evaluation.^10^, ^12^, ^13^, ^18^ In all four studies, MDCT and TEE‐based annulus measurements were compared in the same group of patients. A total of six studies met the inclusion criteria.^19^, ^20^, ^21^, ^22^, ^23^, ^24^ Five studies compared MDCT and 2DTEE for annulus sizing,^19^, ^20^, ^21^, ^22^, ^23^ while one study compared MDCT with 3DTEE‐based annulus sizing.^24^ The characteristics of the included studies are presented in Table 1. All studies had obtained informed written consent from study participants and were approved by the institutional ethical committee.

**Table 1:** Characteristics of the included studies

Study	MDCT equipment	TEE equipment	Type of TEE	MDCT technique	TEE technique	Choice of valve based on		Valve type used	Postprocedural PVR evaluation using	Study results
						MDCT group	TEE group			
Hayashida et al,^19^ 2012	Philips Brilliance 64‐slice MDCT scanner (Philips Medical, Cleveland, Ohio)	Philips iE33 ultrasound system (Philips Medical, Amsterdam, The Netherlands)	2D	Average of three manual measurements in mid‐systole double‐oblique transverse view	Average of three manual measurements from long‐axis end‐systolic view	MDCT, TEE and TTE	TEE and TTE	Edwards SAPIEN (85.7%) with diameter 23,26 and 29 mm and CoreValve (14.3%) with diameter 26, 29, and 31 mm.	NR	Significantly less PVR with addition of MDCT in annular sizing protocol.
Jilaihawi et al,^23^ 2012	Siemens Somatom Cardiac 64 scanner (Siemens Medical Solutions USA Inc, Malvern, Pennsylvania)	NR	2D	Manual measurement in mid‐systole coronal and double‐oblique transverse view	Manual measurement from long‐axis mid‐systolic view	MDCT and TEE	TEE and TTE	Edward SAPIEN 23, 26 mm	TEE	Significantly less PVR with addition of MDCT in annular sizing protocol.
Binder et al,^21^ 2012	Discovery HD 750, GE Healthcare, Waukesha, Wisconsin or Siemens Somatom Definition Flash, Siemens Healthcare, Erlangen, Germany	NR	2D	Manual measurement in mid‐systole, plane NR	Details NR	MDCT and TEE	TEE	Edwards SAPIEN XT 20, 23, 26, 29 mm	TTE	Significantly less PVR with MDCT as compared to 2DTEE.
Hansson et al,^22^ 2013	Second‐generation dual‐source CT system (Siemens Somatom Definition Flash, Siemens Healthcare, Erlangen, Germany)	NR	2D	Manual measurement in mid‐systole double‐oblique transverse view	Average of three to five manual measurements in a mid‐systolic long‐axis view at 120–135°	MDCT	TEE	Edwards SAPIEN or SAPIEN XT 23, 26, 29 mm	TEE	Significantly less PVR with MDCT as compared to 2DTEE.
Casset et al,^20^ 2017	Philips Brilliance 64‐slice MDCT scanner (Philips Medical, Cleveland, Ohio)	Live 2DTEE probe X7‐2t, Philips medical system, Cleveland, Ohio	2D	Manual measurement in mid‐systole double‐oblique transverse view	Manual measurements in long‐axis view at 120°	MDCT, TEE, and TTE	TEE and TTE	Edwards SAPIEN XT 23, 26, 29 mm	TTE	Significantly less PVR with addition of MDCT in annular sizing protocol. No difference in major vascular complications and all‐cause death between the two groups
Wystub et al,^24^ 2019	Revolution CT, GE Healthcare, Milwaukee, Wisconsin	X7‐2t Live 3‐DTEE transducer, Epiq‐7, Philips, The Netherlands	3D	Manual measurements in double‐oblique transverse view	Manual measurements in early systolic long‐axis view at 120°	MDCT	TEE	Edward SAPIEN and Evolut R	TTE	Significantly less PVR with MDCT as compared to 3DTEE.

Abbreviations: 2D = two‐dimensional; 3D = three‐dimensional; MDCT = multidetector computed tomography; NR = not reported; PVR = paravalvular regurgitation; TEE = transesophageal echocardiography; TTE = transthoracic echocardiography.

Echocardiogram‐based and MDCT‐based annulus sizing was done at different time intervals in all studies, and data were analyzed retrospectively, except for one trial. Casset et al^20^ conducted a prospective randomized trial evaluating the addition of MDCT to TEE and TTE‐based annulus measurements on postoperative outcomes. Measurements were recorded in the systolic phase for both groups in all studies. Except for two studies,^22^, ^24^ both MDCT and TEE‐based measurements were available to the operator during the procedure. Valves implanted were exclusively Edward SAPIEN or SAPIEN XT in four studies,^20^, ^21^, ^22^, ^23^ Edward Sapien and CoreValve in one study^19^ and Edward Sapien and Evolut R in another study.^24^ The risk of bias assessment of included studies is presented in Table 2, and the baseline characteristics of the participants of all six studies are presented in Table 3.

**Table 2:** Risk of bias assessment

Study	Random sequence generation	Allocation concealment	Blinding of participants and personnel	Blinding of outcome assessment	Incomplete outcome data	Selective reporting
Randomized studies						
Casset et al^20^	Low risk	Low risk	High risk	High risk	Low risk	Low risk
Nonrandomized studies						
Study	Selection of participants	Confounding variables	Intervention measurements	Blinding of outcome assessment	Incomplete outcome data	Selective outcome reporting
Hayashida et al^19^	High risk	High risk	Low risk	High risk	Low risk	Low risk
Jilaihawi et al^23^	High risk	Unclear risk	Low risk	Low risk	Low risk	Low risk
Binder et al^21^	High risk	Unclear risk	Low risk	High risk	Low risk	Low risk
Hansson et al^22^	High risk	Unclear risk	Low risk	High risk	Low risk	Low risk
Wystub et al^24^	High risk	Unclear risk	Low risk	High risk	Low risk	Low risk

**Table 3:** Baseline characteristics of patients in studies comparing MDCT and TEE‐based annular sizing

Author/Year	Hayashida et al^19^		Jilaihawi et al^23^		Binder et al^21^		Hansson et al^22^		Casset et al^20^		Wystub et al^24^	
	MDCT	2DTEE	MDCT	2DTEE	MDCT	2DTEE	MDCT	2DTEE	MDCT	2DTEE	MDCT	3DTEE
Sample size	175	175	96	40	133	133	58	80	25	25	116	111
Age (y)	83.2 ± 6.4	83.3 ± 6.4	82.4 ± 10.2	84.9 ± 7.2	82 ± 8	81 ± 8	82.6 ± 6	81.2 ± 6.9	83.2 ± 7.8	85.3 ± 6.8	80 ± 6	79 ± 8
Male (%)	52	49.7	45	52.1	57	63	44.8	51.2	48	40	42.2	50.5
BMI, kg/m^2^	26.0 ± 4.2	25.6 ± 4.5	‐	‐	27 ± 6	27 ± 6	25.9 ± 5	25.5 ± 4.4	26.1 ± 4.2	25.4 ± 4.6	28 ± 5.1	28 ± 5.7
NYHA class III/IV (%)	82.6	84	‐	‐	54.13	63.15	77.6	87.5	84	72	71.9	90
Diabetes (%)	22.3	23.4	32.5	27.7	32	35	27.6	25	24	16	40	34.2
Hypertension (%)	70.9	69.7	92.5	85.1	84	79	86.2	85	64	68	‐	‐
COPD (%)	25.7	34.3	50	59.6	38	26	20.7	18.8	‐	‐	32.7	36.1
CAD or PCI (%)	54.9	62.3	30	38.3	‐	‐	36.2	46.3	52	60	51.3	63.1
Previous MI (%)	7.4	14.9	‐	‐	21	30	29.3	32.5	‐	‐	‐	‐
PVD (%)	29.7	30.3	‐	‐	20	19	13.8	20	12	4	‐	‐
Logistic EuroScore	20.1 ± 10.4	24.4 ± 11.5	27.5 ± 14.5	31.2 ± 16.1	‐	‐	18.9 ± 12.6	23.2 ± 16.1	19.5 ± 11.1	23 ± 8.5	15.7 ± 11	16 ± 11
LVEF (%)	‐	‐	61.5 ± 11.8	58.9 ± 14.7	53 ± 14	51 ± 15	‐	‐	53.8 ± 14.1	51.8 ± 10.9	‐	‐
Aortic valve area (cm^2^)	0.64 ± 0.13	0.62 ± 0.16	‐	‐	0.7 ± 0.2	0.7 ± 0.2	0.67 ± 0.19	0.67 ± 0.2	0.7 ± 0.2	0.6 ± 0.1	‐	‐
Mean aortic gradient (mm Hg)	48.3 ± 16.5	47.0 ± 16.5	44.5 ± NR	43 ± NR	42 ± 18	38 ± 15	66.1 ± 27.6	71 ± 28.5	46.2 ± 19.1	50.3 ± 13.4	‐	‐
Transfemoral route (%)	54.28*	58.28	87.5	82.3	74	71	46.5	22.5	100	100	100	100
Transapical route (%)	21.14*	31.42	12.5	17.7	18	28	51.7	77.5	‐	‐	‐	‐
Annulus diameter (mm)	NR	NR	23.2 ± 2.1	22.6 ± 2.2	21.5 ± 2.2	22.5 ± 3	22.1 ± 2.3	22.4 ± 1.6	21 ± 1.7	20.4 ± 1.8	21.4 ± 2.3	21.2 ± 2.2
Mean prosthesis size	23 mm: 26% 26 mm: 60.7% 2 9 mm: 13.3%*	23 mm: 46.3% 26 mm: 51.7% 29 mm: 2%	23 mm: 62.5% 26 mm: 37.5%	23 mm: 57.5% 26 mm: 42.5%	20 mm: 0.8% 23 mm: 26.3% 26 mm: 45.9% 29 mm: 27.1%*	20 mm: 1.5% 23 mm: 30.8% 26 mm: 51.1% 29 mm: 16.5%	23 mm: 15.5% 26 mm: 51.7% 29 mm: 32 7%*	23 mm: 45% 26 mm: 55% 29 mm: 0%	24.6 ± 1.7*	23.7 ± 1.3	23 mm: 19% 26 mm: 36.2% 29 mm: 44.8%*	23 mm: 28.4% 26 mm: 45% 29 mm: 25.7% 3 mm: 0.9%

Abbreviations: BMI = body mass index; CAD = coronary artery disease; COPD = chronic obstructive pulmonary disease; 2D = two‐dimensional; 3D = three‐dimensional; LVEF = left ventricular ejection fraction; MDCT = multidetector computed tomography; MI = myocardial infarction; NR = not reported; NYHA = New York Heart Association; PCI = percutaneous coronary intervention; PVD = peripheral vascular disease; TEE = transesophageal echocardiography.

Data presented as number, percentage, or Mean ± Standard Deviation.

* Significant difference in prosthesis size between MDCT and TEE groups.

Meta‐analysis was carried out for five studies comparing outcomes following MDCT and 2DTEE‐based annulus measurements.^19^, ^20^, ^21^, ^22^, ^23^ The age of the included patients was >70 years in all studies. Male gender percentage ranged from 44.8% to 63%. The percentage of patients with New York Heart Association (NYHA) score of III/IV were 54.13%‐87.5%. Study and control groups were matched on most baseline characteristics in all cohorts. A significantly larger prosthesis was utilized in patients with MDCT‐based annulus measurements as compared to those with 2DTEE‐based annulus measurements, in four of the five studies.^19^, ^20^, ^21^, ^22^

**Figure 2:**
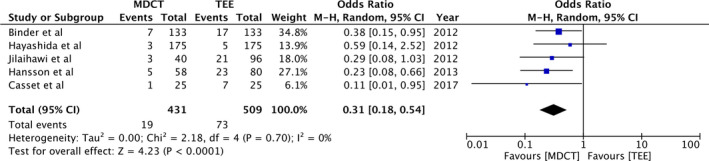
Forest plot of more than moderate PVR

**Figure 3:**
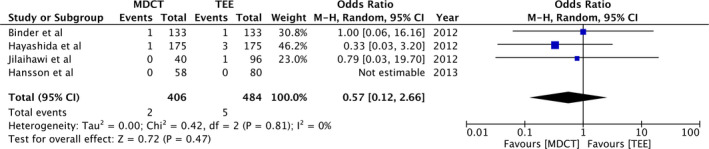
Forest plot of annulus rupture

**Figure 4:**
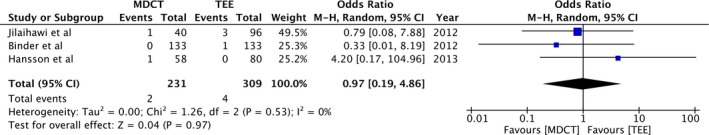
Forest plot of procedural mortality

**Figure 5:**
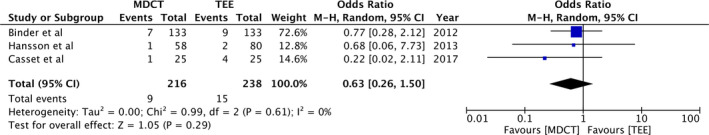
Forest plot of 30‐d mortality

Pooled analysis of 431 participants in the study group and 509 participants in the control group demonstrated that MDCT‐based annulus sizing results in significantly lower incidence of more than moderate PVR (OR: 0.31, 95%CI: 0.18‐0.54, *P* < .0001; *I^2^* = 0%) (Figure 2). Details on the incidence of annulus rupture were reported by four studies. Meta‐analysis indicated no statistically significant difference in annulus rupture with MDCT or 2DTEE‐based annulus sizing (OR: 0.57, 95%CI: 0.12‐2.66, *P* = .91; *I* ^2^ = 0%) (Figure 3). Data on procedural mortality and 30‐day mortality were reported by three studies. Procedural mortality in the MDCT group was 0.86% while in the 2DTEE group was 1.29%, with pooled analysis demonstrating no significant difference (OR: 0.97, 95%CI: 0.19‐4.86, *P* = .97; *I* ^2^ = 0%) (Figure 4). The incidence of 30‐day mortality in patients with MDCT‐based annulus sizing (4.16%) and 2DTEE‐based sizing (6.30%) was also not significantly different (OR: 0.63, 95%CI: 0.26‐1.50, *P* = .29; *I* ^2^ = 0%) (Figure 5). On sensitivity analysis, there was no change in significance of the results on exclusion of any study in any of the pooled analysis.

### 3.1 MDCT vs 3DTEE

In the retrospective study of Wystub et al,^24^ MDCT was used for annulus sizing in 116 patients and 3DTEE was utilized in 111 patients. There was no significant difference in the baseline characteristics of the two groups. Significantly larger valves were used in the MDCT group as compared to the 3DTEE group (Table 3). A significantly higher number of patients in the MDCT group (57.6%) did not have PVR as compared to the TEE group (35.3%; *P* = .016). There was no difference in 30‐day mortality between the two groups (3.4% in MDCT group vs 0.9% in 3DTEE group, *P* = .181).

## 4 DISCUSSION

Of the two types of aortic valvular regurgitation, central regurgitation is usually seen in diseased native valves whereas PVR is a complication seen only after TAVR.^25^ Since the native valve is still in situ when the prosthesis is placed over the biological tissue, an incomplete seal may remain, thereby resulting in PVR.^25^ Despite a technological improvement in devices to provide an efficient seal between the aortic annulus and the implanted prosthesis, the incidence of PVR is as high as 23.8% post TAVR.^26^ The PARTNER trial has demonstrated that even mild PVR is associated with an increased risk of late mortality.^27^ Similar results have been obtained by other studies wherein more than moderate PVR was found to be a strong predictor of in‐hospital death.^3^, ^28^ While redilatation or implantation of valve‐in‐valve may be attempted as a corrective measure for PVR, steps for prevention of PVR are necessary for good clinical outcomes.^29^

Complications like PVR after TAVR are usually the result of inappropriate prosthesis size selection. While the annulus can be directly inspected for sizing in surgical aortic valve replacement (SAVR), selecting the prosthesis size is completely dependent on imaging studies in TAVR.^30^ Traditionally, 2DTEE was the method of choice for annulus sizing.^31^ However, with the introduction of MDCT, dependency on echocardiography for annulus sizing has been reduced in many centers worldwide.^11^ The higher spatial resolution of MDCT provides accurate annulus dimensions resulting in more appropriate prosthesis size selection.^20^ On the other hand, measurements obtained by 2DTEE are frequently undersized resulting in implantation of a smaller prosthesis.^19^ In most of the included studies of this review, a significantly larger prosthesis was selected for implantation in the MDCT group as compared to the TEE group.

Despite MDCT becoming the gold standard imaging for annulus sizing, the requirement of contrast media is a significant limitation especially in patients with severe renal impairment.^32^ An estimated 7%‐10.5% of TAVR patients have been found to have MDCT contrast‐related kidney injury.^33^ With around 70% of the TAVR population having preoperative renal disease, TEE may still be an alternative imaging modality for such patients.^34^ It may also be useful in individuals with iodine allergy, centers with high patient load or due to economic constraints.^20^ In the absence of dynamic information by MDCT, TEE also yields better temporal resolution that aids in tracing calcified nodules and identification of mobile components.^20^ In the face of such differences, it is important to analyze the differences in clinical outcomes following MDCT and TEE‐based annulus measurements for TAVR.

To date, a total of six studies have compared clinical outcomes following MDCT and TEE‐based measurements for TAVR and most of them have utilized 2DTEE in the echocardiography group. The results of our analysis indicate that the use of MDCT for annulus sizing is associated with an estimated 69% decrease in the incidence of more than moderate PVR as compared to 2DTEE‐based sizing. The significant difference in the incidence of PVR between the two groups is largely attributed to the underestimation of annulus size by 2DTEE. Dashkevich et al^30^ have demonstrated poor correlation between intra‐operative annulus measurements and 2DTEE‐based dimensions with TEE frequently underestimating the aortic annulus size. Our results failed to demonstrate any difference in the incidence of annulus rupture as well as procedural and 30‐day mortality between the two imaging modalities. This could be attributed to the rare occurrence of these events and the limited number of studies with small sample size of the cohorts in our analysis. Further, larger studies may detect differences, if any, for these outcome variables.

To overcome the limitations of 2DTEE, 3DTEE has been introduced as an alternative to MDCT‐based annulus sizing.^11^ Advances in 3DTEE technology with a multiplanar reconstruction of the aortic root and outflow tract as well as annulus sizing software have improved the efficiency of this imaging modality.^35^ In a recent meta‐analysis, Rong et al^11^ have demonstrated a strong correlation between MDCT‐based and 3DTEE‐based measurements for TAVR. However, to date, only one study has compared the incidence of complications following MDCT vs 3DTEE‐based annulus sizing. Wystub et al,^24^ comparing two cohorts of TAVR patients treated at different time intervals, found a reduced incidence of PVR in the MDCT group. Similar to 2DTEE, underestimation of annulus size resulting in smaller prosthesis was described as the probable reason for the difference in PVR.^24^

The results of our review are to be interpreted with the following limitations. Foremost, a limited number of studies with small sample size were available for analysis. Only one study was analyzed for MDCT vs 3DTEE‐based annulus sizing. Secondly, only one prospective randomized study has compared MDCT and TEE for annulus sizing. All remaining studies compared cohorts evaluated by either imaging modality at different time intervals. The inherent bias of retrospective observational studies may have skewed the overall results. Thirdly, there was significant variation between studies in terms of differences in types of prosthesis used, prosthesis size, method of evaluation for postoperative PVR (TEE and TTE), etc Fourthly, prosthesis sizing was not singularly dependent on MDCT or TEE in most of the studies, but was influenced by operator preferences, anatomical factors, and other imaging studies as well. Lastly, we could not analyze all postoperative outcomes like the incidence of vascular complications and pacemaker implantation, due to the paucity of data. Long‐term mortality data were also not available from the included studies for a pooled analysis.

This is the first systematic review and meta‐analysis evaluating outcomes after MDCT vs TEE‐based annulus sizing for TAVR. After the pooling of data of more than 800 patients, our results indicate that the use of MDCT against 2DTEE is associated with a significantly reduced incidence of more than moderate PVR after TAVR. However, there seems to be no difference in annulus rupture, procedural, and 30‐day mortality with either imaging modality. Further studies are required to provide evidence on postoperative outcomes following MDCT or 3DTEE‐based annulus sizing.

## Supporting information

Table S1. Search protocol and PubMed results.
