# Supplementary material for: Comparison of postoperative outcomes following multidetector computed tomography based vs transesophageal echocardiography based annulus sizing for transcatheter aortic valve replacement: A systematic review and meta‐analysis
Source: Echocardiography. 2020 Sep 23;37(10):1617–26. doi: 10.1111/echo.14684 (PMC7702059; doi:10.1111/echo.14684)
Supplement: Supplementary file 1 — Table S1. Search protocol and PubMed results. [file ECHO-37-1617-s001.docx]

Supplementary document 1: Search protocol and PubMed results

| Search no | Search terms | Number of records |
| --- | --- | --- |
| 1 | Search (((Multidetector Computed Tomography) OR Transoesophageal Echocardiography) AND transcatheter aortic valve implantation) AND clinical outcomes | 112 |
| 2 | Search ((Computed Tomography) OR Echocardiography) AND paravalvular leak | [500](https://www.ncbi.nlm.nih.gov/pubmed/?cmd=HistorySearch&querykey=6) |
| 3 | Search ((Multidetector Computed Tomography) OR Transoesophageal Echocardiography) AND paravalvular regurgitation | [309](https://www.ncbi.nlm.nih.gov/pubmed/?cmd=HistorySearch&querykey=5) |
| 4 | Search ((MDCT) OR TEE) AND transcatheter aortic valve replacement | [222](https://www.ncbi.nlm.nih.gov/pubmed/?cmd=HistorySearch&querykey=4) |
| 5 | Search ((Computed Tomography) OR Echocardiography) AND transcatheter aortic valve implantation | [2872](https://www.ncbi.nlm.nih.gov/pubmed/?cmd=HistorySearch&querykey=3) |
| 6 | Search ((Multidetector Computed Tomography) OR Transoesophageal Echocardiography) AND transcatheter aortic valve replacement | [837](https://www.ncbi.nlm.nih.gov/pubmed/?cmd=HistorySearch&querykey=1) |
| 7 | Search (((Multidetector Computed Tomography) OR Transoesophageal Echocardiography) AND transcatheter aortic valve implantation) AND clinical outcomes | [112](https://www.ncbi.nlm.nih.gov/pubmed/?cmd=HistorySearch&querykey=7) |
